# Cervical human papillomavirus infection among young women engaged in sex work in Phnom Penh, Cambodia: prevalence, genotypes, risk factors and association with HIV infection

**DOI:** 10.1186/1471-2334-12-166

**Published:** 2012-07-28

**Authors:** Marie-Claude Couture, Kimberly Page, Ellen S Stein, Neth Sansothy, Keo Sichan, John Kaldor, Jennifer L Evans, Lisa Maher, Joel Palefsky

**Affiliations:** 1 University of California San Francisco, Global Health Sciences, 50 Beale street, Suite 1200, San Francisco, CA, 94105, USA; 2 National Institute for HIV, AIDS, Dermatology and STDs, Phnom Penh, Cambodia; 3 Cambodian Women’s Development Agency, Phnom Penh, Cambodia; 4 The Kirby Institute, University of New South Wales, Sydney, Australia

## Abstract

**Background:**

Although cervical cancer is the leading cancer in Cambodia, most women receive no routine screening for cervical cancer and few treatment options exist. Moreover, nothing is known regarding the prevalence of cervical HPV or the genotypes present among women in the country. Young sexually active women, especially those with multiple sex partners are at highest risk of HPV infection. We examine the prevalence and genotypes of cervical HPV, as well as the associated risk factors among young women engaged in sex work in Phnom Penh, Cambodia.

**Methods:**

We conducted a cross-sectional study among 220 young women (15–29 years) engaged in sex work in different venues including brothels or entertainment establishments, and on a freelance basis in streets, parks and private apartments. Cervical specimens were collected using standard cytobrush technique. HPV DNA was tested for by polymerase chain reaction (PCR) and genotyping using type-specific probes for 29 individual HPV types, as well as for a mixture of 10 less common HPV types. All participants were also screened for HIV status using blood samples. Multivariate logistic regression analyses were conducted to assess risk factors for any or multiple HPV infection.

**Results:**

The prevalence of cervical HPV 41.1%. HPV 51 and 70 were the most common (5.0%), followed by 16 (4.6%), 71 (4.1%) and 81 (3.7%). Thirty-six women (16.4%) were infected with multiple genotypes and 23.3% were infected with at least one oncogenic HPV type. In multivariate analyses, having HIV infection and a higher number of sexual partners were associated with cervical HPV infection. Risk factors for infection with multiple genotypes included working as freelance female sex workers (FSW) or in brothels, recent binge use of drugs, high number of sexual partners, and HIV infection.

**Conclusions:**

This is the first Cambodian study on cervical HPV prevalence and genotypes. We found that HPV infection was common among young FSW, especially among women infected with HIV. These results underscore the urgent need for accessible cervical cancer screening and treatment, as well as for a prophylactic vaccine that covers the HPV subtypes present in Cambodia.

## Background

Cervical cancer is the third most common cause of cancer among women worldwide, and it is more frequent in developing countries where population-level screening programs are generally unavailable [1]. In Cambodia, cervical cancer is the most common form of cancer among women with an incidence (age-standardized rate ASR) of 27.4 per 100 000, higher than in other Southeast Asian countries [1]. Few, if any, Cambodian women receive screening for cervical cancer and few treatment options exist [2], likely contributing to a high mortality rate of 16.2 per 100,000 (estimated age-standardized rate ASR) [1].

Genital human papillomavirus (HPV), the most common sexually transmitted infection (STI) worldwide, is the key causal agent in cervical cancer [3,4]. More than 100 different HPV genotypes have been identified, which are divided into “high-risk” (HR) and “low-risk” (LR) types on the basis of their oncogenic potential [5]. Most HPV infections are transient and clinically unapparent. However, persistent infection with cervical HR-HPV genotypes can lead to the development of cervical cancer pre-malignant lesions that may evolve into invasive cervical cancer [5].

Cervical HPV prevalence and genotypes differ substantially from one population to another worldwide. Although cervical cancer is the leading cancer in Cambodia, nothing is known regarding the prevalence of HPV infection or the genotypic distribution in the country. As in other parts of the world, epidemiological studies in Southeast Asia has shown that the distribution of cervical HPV genotypes differ across populations, and oncogenic genotypes other than HPV-16 and −18 are frequent [6-10]. Two commercial vaccines targeting oncogenic HPV-16 and −18 have been shown to be efficacious in reducing both chronic HPV infection, and cervical and vulvovaginal disease related to these genotypes in women [11-13]. These two vaccines have been introduced in several countries and are increasingly becoming available in resource-constrained settings. However, their impact on prevention of HPV infection and cervical cancer may differ regionally, according to the genotype distribution. Accurate epidemiological data on HPV prevalence and genotypes in specific populations will assist public health authorities to plan for immunization implementation, and to guide future vaccine development.

Also of importance in planning clinical and public health responses is an understanding of the factors associated with acquiring cervical HPV infection. Factors detected so far include higher number of sexual partners, early age at first sexual intercourse, smoking, oral contraceptive use, inconsistent condom use, and other STI infections, including HIV [14-17]. The literature has shown that HIV-seropositive women are at greatly increased risk for development of pre-malignant lesions and invasive cervical cancer compared to HIV-seronegative women [18-20]. Moreover, several studies have reported that the prevalence of single or multiple HPV infections and HPV viral load was higher among women infected with HIV [19,21,22]. Women engaged in sex work have multiple sex partners, and are consequently at higher risk of both HIV and HPV infection [6,9,23,24]. Understanding the determinants of cervical HPV infection is an essential step in developing effective interventions for the prevention of infection, and ultimately cervical cancer.

To date, nothing is known regarding cervical HPV prevalence, genotypic distribution, or risk factors in the Cambodian female population. To inform policy and guide further research, we undertook a study to examine cervical HPV genotypes and the associated risk factors among young FSW working in Phnom Penh, Cambodia.

## Methods

### Study setting

Cross-sectional data were obtained from a single baseline visit, in the Young Women’s Health Study 2 (YWHS-2). This prospective study, conducted from August 2009 to August 2010, investigated HIV/STI, sexual behaviors and drug use among women engaged in sex work in Phnom Penh, Cambodia; methods described previously in detail [25]. In brief, women working in brothels, entertainment establishments or on a freelance basis were enrolled in a one-year study, with quarterly follow-up visits to assess HIV and HPV infections and associated risks. The study was undertaken by a multi-disciplinary collaborative prevention research group of academic, governmental and community HIV prevention specialists from the U.S., Australia, and Cambodia.

### Study population and recruitment

A convenience sample of women engaged in sex work was recruited through informational meetings, neighborhood outreach, and referrals by participants. Inclusion criteria for the study were: being female, aged 15–29 years, ethnic Cambodian, reporting at least two different sexual partners in the last month *or* engagement in transactional sex (sex in exchange for money, goods, services, or drugs) in the last three months. Eligible women who were interested in the study were invited to a community location used by various sex-worker organizations, where they were provided with comprehensive study information. A group informed consent process including oral review of the written informed consent, with explanations and opportunities for questions and answers, was conducted. Women who consented to participate were provided with appointment cards to present to the YWHS clinic site. Free transportation was offered to facilitate attendance at scheduled study visits. All women negative for HIV and not pregnant were offered a three-dose regimen of a HPV quadrivalent vaccine (Gardasil^TM^).

### Data collection

A structured paper questionnaire was administered in Khmer by trained interviewers in a private room covering socio-demographic characteristics, occupational and sexual risk, alcohol and drug use exposures. Client-centered risk reduction counseling was provided, and participants were offered testing for HIV and HPV. HIV results were disclosed to participants and women infected with HIV received counseling and were referred for free medical evaluation and treatment, where indicated.

### HPV and HIV testing

Cervical specimens were collected by a medical doctor using a standard cytobrush technique and transported to University of California at San Francisco for HPV testing. HPV DNA was tested by polymerase chain reaction (PCR) and genotyping was conducted using type-specific probes [21,26]. MY09/MY11 primer and probe method and Amplitaq Gold were used to amplify HPV sequences[15,27]. Beta-globin primers were used as an internal positive control for the presence of human DNA. As a negative control, amplification of the reaction mixture was performed with all components except the target DNA. Samples were dot blotted and probed for HPV DNA using a chemiluminescent procedure with a consensus probe mixture. Samples determined to be consensus-probe positive will then were reanalyzed for the presence of 29 individual HPV types, as well as for a mixture of 10 less common HPV types. Samples were classified as HPV-positive or HPV-negative based on the results of the consensus probe. If a sample was consensus probe-positive but negative for all 39 HPV types, it was considered to have an unknown HPV type. HPV infection was classified as high risk (types 16, 18, 26/69, 31, 33, 35, 39, 45, 51, 52, 53, 56, 58, 59, 66, 68, 73 or 82) or low risk (types 6, 11, 32/42, 54, 61, 62, 67, 70, 71, 72, 81, 83 or 84) based on strength of association of specific HPV types with invasive cervical cancer[28].

HIV serology with venous blood was performed using two rapid tests; Uni-Gold Recombigen ^(TM)^ HIV rapid HIV test (Trinity Biotech USA, Jamestown, NY) and the Clairview HIV 1/2 STAT-PAK (Inverness Medical Diagnostics, Waltham, MA). HIV-positive and discordant samples were confirmed by HIV-1 immunoblot. HIV specimen testing occurred at the Cambodian National Institute of Public Health (NIPH) laboratory.

### Ethical review

The study protocol was reviewed and approved by the Cambodian National Ethical Committee, the Committee on Human Research at UCSF and the University of New South Wales Human Research Ethics Committee.

### Measures

Outcome variables were “any HPV infection” and “multiple HPV infection” (two or more genotypes). Sociodemographic variables included age, marital status, education and number of children. Based on sex work activities in the past 30 days, women were classified as working in either 1) entertainment establishments (beer gardens, bars, karaoke, and night clubs) or 2) working as freelance FSW in streets and parks or in brothels. The promulgation of anti-sex-trafficking and sexual exploitation laws enacted in 2008 has resulted in the closure of brothels and the displacement of many FSW to entertainment establishments or outdoor settings. At the time this study was conducted (2009–2010) most of the brothels were closed and only five women reported working in brothels. Drug and alcohol use variables included recent (last 3 months) amphetamine-type stimulants (ATS) use, recent binge use of drug, number of days of alcohol use and the number of days drunk (past month) [29]. Recent binge of drug was assessed by asking participants if they had used any drugs for more than 48 hours continuously without sleep in the last 3 months. Variables related to sexual behaviors included age of first sexual intercourse, number of sexual partners (last month), number of new sexual partners (last month) and condom use with last paying and non-paying partner. Condom use was classified as “consistent” if the participant reported always using a condom. Previously-diagnosed STI were assessed by asking participants if a doctor, nurse, or other health care provider had ever told them that they had been diagnosed with a sexually transmitted infection.

### Statistical analyses

Data were double entered into a database software (Access, Microsoft Corp., Redmond, WA, USA) and transferred into STATA 11.0 (STATA, College Station, TX, USA) for statistical analyses. Bivariate logistic regression analyses were performed to identify associations between socio-demographics, drug and alcohol use, sexual behaviors, HIV and STI infections and HPV outcomes: 1) “any HPV infection”, and 2) “multiple HPV infection (≥2)”. Multivariate logistic regression models were built according to a conceptual framework where variables were entered as groups in steps [30]: 1) sociodemographics; 2) drug and alcohol use; 3) sexual behaviors; 4) HIV and STI infections. First, sociodemographic variables (group 1) with a level of significance in bivariate analysis of p<0.20 were included in the model in a stepwise backward approach, leading to our first model including only sociodemographic variables associated with the outcome at p<0.10. Other variables were then entered into the model in groups using the same stepwise backward procedure, adjusting for the variable included in the precedent model. In addition, variables excluded in the earlier steps of the modeling were added in the final model to assess their associations with the outcome variable in the presence of the other variable or their possible confounding effect. Only variables significant at p<0.05, important confounders and potential explanatory variables were retained in the final model. Analyses were performed using STATA 11.0 (STATA, College Station, TX).

## Results

**Figure 1:**
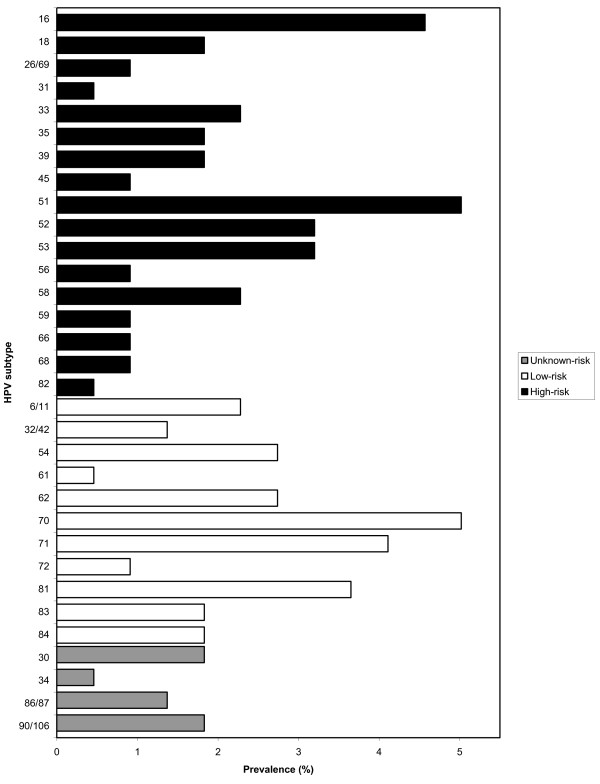
Prevalence of different cervical HPV genotypes among young women engaged in sex work participating in the Young Women’s Health Study in Phnom Penh, Cambodia.

**Figure 2:**
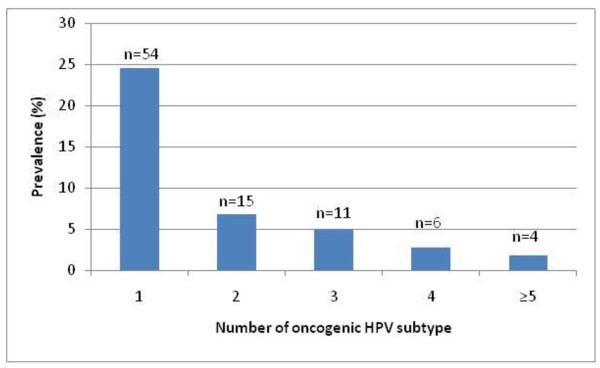
Prevalence of infection with multiple cervical HPV genotypes among young women engaged in sex work participating in the Young Women’s Health Study in Phnom Penh, Cambodia.

The prevalence of HIV was 15.8% among 220 participants. Cervical HPV DNA was detected in 41.1% (90/220) of women; HPV prevalence was 34.4% (64/186) among HIV negative women and 78.8% (26/33) among those who were HIV positive. Almost a quarter (23.2%) were infected with at least one oncogenic high-risk (HR-HPV) genotype and 17.7% with at least one low risk subtype (LR-HPV). HPV-51 (5.0%; 11/220) and HPV-16 (4.6%; 10/220) were the most common HR-HPV genotypes present, followed by HPV-52 and HPV-53 (each 3.2%; 7/220). Among those infected with LR-HPV genotypes, HPV-70 was the most common (5.0%; 11/220), followed by HPV-71 (4.1%; 9/220) and HPV-81 (3.7%; 8/220) (Figure 1). Thirty-six women (16.8%) were infected with multiple cervical HPV genotypes and four women (1.8%) had five or more different genotypes (Figure 2).

Table 1 shows sociodemographic characteristics, drug and alcohol use, sexual behaviors, HIV and STI infections and their associations with any or multiple cervical HPV infection. Participants’ median age was 26 years (IQR 22, 28), almost a quarter (22.4%) had no education, and 41.6% were widowed, divorced or separated. Women reported working in sex work for a median of 3.5 years (interquartile range (IQR 2.5, 6.3) and most (68.0%) were working in the entertainment sector. Alcohol and ATS use were prevalent. Median number of days drinking and being drunk in the last month were 15 (IQR 4, 29) and 3 (IQR 0, 10) days, respectively), and the number of days of drinking was inversely associated with prevalent HPV (p ≤ 0.05). Women who reported recent ATS use, including binge use, had significantly higher risk of cervical HPV infection (any or multiple). Women reported a median of 6 (IQR 4, 15) sexual partners in the last month and many were new (median = 3; IQR 1, 6). Condom use during the last sexual intercourse was high with paying partners (90.8%), but low with non-paying partners (16.8%), mainly boyfriends and husbands. Almost sixty percent (58.7%) reported ever been diagnosed with a STI. The prevalence of any or multiple cervical HPV infection was significantly (p ≤ 0.01) higher among women who worked as freelance in streets/parks or in brothels (58.6% for any HPV and 33.8% for multiple infection) compared with those working in entertainment establishments (32.9% and 8.7%). FSW who reported more sexual partners or new partners (≥16 compared to less than 16) were more likely to have any or multiple cervical HPV infection. The prevalence of HPV (any or multiple) was significantly higher among HIV-infected women and those who reported having had a previous STI diagnosis (Table 1).

**Table 1:** Characteristics and prevalence of any and multiple cervical HPV infection among women engaged in sex work participating in the Young Women’s Health Study in Phnom Penh, Cambodia

**Characteristic**	**Prevalence of characteristic**	**Prevalence of any HPV infection**		**Prevalence of multiple HPV (≥2 genotypes) infection**	
	**N (%)**	**N (%)**	**OR (95% CI)**	**N (%)**	**OR (95% CI)**
**Overall**	220	90 (41.1)		37 (16.8)	
***Sociodemographics***					
**Age (years):** Median 26; IQR (22, 28)		1.00 (0.93-1.08)^†^			1.04 (0.94-1.15) ^†^
16-18	14 (6.4)	5 (35.7)	1.0	1 (7.1)	1.0
19-24	71 (32.4)	29 (40.9)	1.20 (0.36-3.95)	11 (15.5)	2.38 (0.28-20.11)
25-29	134 (42.8)	56 (41.8)	1.31 (0.42-4.13)	25 (18.5)	2.95 (0.37-23.64)
**Education (years):** Median 4; IQR (1, 6)		0.93 (0.85-1.02) ^†^			0.93 (0.82-1.05)
None	49 (22.4)	23 (46.9)	1.0	12 (24.0)	1.0
Primary (1–6 years)	124 (56.6)	54 (43.6)	0.85 (0.44-1.67)	20 (16.0)	0.60 (0.27-1.35)
Secondary (7+ years)	46 (21.1)	13 (28.3)	0.46 (0.20-1.08)	5 (11.1)	0.40 (0.13-1.23)
**Marital status**					
Never married	44 (20.1)	22 (50.0)	1.0	8 (17.8)	1.0
Married-living together	84 (38.4)	36 (35.2)	0.75 (0.36-1.56)	18 (21.4)	1.26 (0.50-3.18)
Widowed/Divorced/Separated	91 (41.6)	32 (42.9)	0.54 (0.26-1.13)	11 (12.1)	0.64 (0.24-1.71)
**Number of child:** Median 1; IQR (0, 1)		0.80 (0.59-1.09) ^†^			1.08 (0.74-1.58) ^†^
0	89 (40.6)	43 (48.3)	1.0	16 (17.8)	1.0
1	78 (35.6)	28 (35.9)	0.60 (0.32-1.15)	11 (14.1)	0.76 (0.33-1.75)
More than 2	52 (23.7)	19 (36.5)	0.54 (0.26-1.13)	10 (19.2)	1.10 (0.46-2.64)
**Type of sex venue (last 30 days)**					
Entertainment	149 (68.0)	49 (32.9)	1.0	13 (8.7)	1.0
Freelances/brothels	70 (32.0)	41 (58.6)	2.89 (1.61-5.18)**	24 (33.8)	5.34 (2.52-11.33)**
***Drug and alcohol use***					
**ATS use (last 3 months)**					
No	160 (72.7)	53 (33.1)	1.0	18 (11.3)	1.0
Yes	60 (27.3)	37 (62.7)	3.40 (1.82-6.32)**	19 (31.7)	3.66 (1.76-7.60)**
**Binge of drugs (last 3 months)**					
No	179 (81.7)	65 (36.3)	1.0	22 (12.3)	1.0
Yes	40 (18.3)	25 (62.5)	2.93 (1.44-5.94)**	15 (36.6)	4.12 (1.89-8.95)**
**Number of days of drinking alcohol (last month):**		0.98 (0.95-0.99) ^†^*			0.97 (0.94-0.99) ^†^*
0 – 4	57 (26.5)	32 (56.1)	1.0	16 (27.6)	1.0
5 – 19	57 (25.9)	20 (35.1)	0.40 (0.19-0.86) ^†^*	8 (14.3)	0.44 (0.17-1.12)
> 20	105 (47.7)	38 (36.2)	0.45 (0.24-0.88) ^†^*	13 (12.3)	0.37 (0.16-0.83)*
**Number of days drunk (last month):**		0.99 (0.95-1.03)			0.98 (0.93-1.03)
0 – 4	123 (56.2)	57 (46.3)	1.0	27 (21.8)	1.0
5 – 19	72 (32.9)	22 (30.6)	0.51 (0.28-0.94) ^†^*	6 (8.3)	0.33 (0.13-0.83)*
> 20	24 (11.0)	11 (45.8)	0.98 (0.41-2.36)	4 (16.7)	0.72 (0.23-2.28)
***Sexual behaviors***					
**Age at first sexual intercourse**					
≤ 15	37 (16.9)	19 (51.4)	1.0	8 (21.6)	1.0
> 15	180 (82.2)	70 (38.9)	1.00 (0.97-1.04)	28 (15.5)	1.02 (0.99-1.05)
**Number of sexual partners (last month):**		1.06 (1.03-1.09) ^†^**			1.06 (1.03-1.09) ^†^**
≤5	99 (45.2)	31 (31.3)	1.0	9 (9.1)	1.0
6 – 15	71 (32.4)	27 (38.0)	1.35 (0.71-2.55)	8 (11.3)	1.27 (0.46-3.47)
≥ 16	49 (22.4)	32 (65.3)	4.13 (2.00-8.53)**	20 (40.0)	6.67 (2.74-16.21)**
**Number of new partners (last month):**		1.09 (1.04-1.14) ^†^**			
0	43 (19.6)	16 (37.2)	1.0	4 (9.3)	1.0
1-5	120 (54.8)	40 (33.3)	0.85 (0.41-1.74)	18 (14.8)	1.70 (0.54-5.35)
>6	56 (25.6)	34 (60.1)	2.61 (1.15-5.91)*	25 (26.8)	3.57 (1.09-11.69)*
**Condom use with last paying partner**					
Consistent (always)	197 (90.8)	83 (42.1)	1.0	33 (16.7)	1.0
Inconsistent	20 (9.2)	6 (30.0)	0.59 (0.22-1.60)	3 (15.0)	0.88 (0.24-3.18)
**Condom use with last non- paying partner**					
Consistent (always)	18 (16.8)	7 (38.9)	1.0	2 (11.1)	1.0
Inconsistent	89 (83.2)	34 (38.2)	0.97 (0.34-2.75)	12 (13.5)	1.25 (0.25-6.12)
***HIV and STI infections***					
**HIV result**					
Negative	186 (84.9)	64 (34.4)	1.0	22 (11.8)	1.0
Positive	33 (15.1)	26 (78.8)	7.08 (2.91-17.20)**	15 (44.1)	5.89 (2.62-13.23)**
**Previously diagnosed STI**					
No	91 (41.7)	29 (31.9)	1.0	12 (13.2)	1.60 (0.76-3.38)
Yes	127 (58.3)	61 (48.)	1.98 (1.13-3.47)*	25 (19.5)	

† = variable use as continuous * = p ≤ 0.05 ** = p ≤ 0.01.

Table 2 shows multivariate logistic regression analyses of risk factors for having any or multiple cervical HPV infection. Of the sociodemographic and drug and alcohol use variables, only being a freelance or brothel-based FSW and recent ATS use were significantly (p ≤ 0.05) associated with having any HPV infection. However, these associations were not non-significant when number of sexual partners (last month) was entered in the model. In the final multivariate model, higher number of sexual partners (AOR 1.05; 95% CI: 1.01-1.09) and being infected with HIV (AOR 7.96; 95% CI: 2.80-22.65) were the only variables independently and significantly associated with having any cervical HPV infection. Working as a freelance FSW or in a brothel was significantly associated with multiple cervical HPV infection types. However the association was attenuated when recent binge use of drugs and number of sexual partners were entered into the model. Reporting recent binge use of drug was independently associated with having multiple cervical HPV infection (AOR 3.45; 95% CI: 1.32-9.03), as was having more sexual partners (AOR 1.09; 95% CI: 1.03-1.16) and having HIV infection (AOR 5.25; 95% CI: 1.95-14.14).

**Table 2:** Variables independently associated with prevalence of any (A) or multiple (B) cervical HPV infection among women engaged in sex work participating in the Young Women’s Health Study in Phnom Penh, Cambodia (n = 220)

	**A. Any HPV infection**			
	**Model 1**	**Model 2**	**Model 3**	**Final model**
Variables	AOR (95% CI)	**AOR (95% CI)**	**AOR (95% CI)**	**AOR (95% CI)**
Sociodemographics				
***Number of child***	0.77 (0.56-1.05)^†^	0.84 (0.61-1.16)^†^	0.80 (0.57-1.11)^†^	0.70 (0.49-1.01)^†^
**Type of sex venue (last 30 days)**				
Entertainment/others	1.0	1.0	1.0	1.0
Freelances/brothels	3.00 (1.66-5.43)**	2.11 (1.08-4.12)*	1.30 (0.60-2.84)	0.77 (0.32-1.88)
**Drugs and alcohol use**				
**ATS use (last 3 months)**				
No		1.0	1.0	1.0
Yes		2.27 (1.11-4.62)*	1.85 (0.88-3.89)	1.85 (0.83-4.12)
***Sexual behaviors***				
**Number of sexual partners (last month)**			1.05 (1.01-1.08)^†^*	1.05 (1.01-1.09)^†^*
*HIV and STI infections*				
**Previously diagnosed STI**				
No				1.0
Yes				1.55 (0.82-2.92)
**HIV result**				
Negative				1.0
Positive				7.96 (2.80-22.65)**
	**B. Multiple HPV infection (≥2 genotypes)**			
	**Model 1**	**Model 2**	**Model 3**	**Final Model**^**§**^
Variables	OR (95% CI)	**AOR (95% CI)**	**AOR (95% CI)**	**AOR (95% CI)**
Sociodemographics				
**Type of sex venue (last 30 days)**				
Entertainment/others	1.0	1.0	1.0	1.0
Freelances/brothels	5.34 (2.52-11.33)**	4.18 (1.90-9.21)**	3.56 (1.44-8.82)**	3.05 (1.05-8.84)*
**Drugs and alcohol use**				
**Binge of drugs (last 3 months)**				
No		1.0	1.0	1.0
Yes		2.58 (1.12-5.96)*	2.66 (1.09-6.51)**	3.45 (1.32-9.03)*
***Sexual behaviors***				
**Number of sexual partners (last month)**			1.07 (1.02-1.13)^†^**	1.09 (1.03-1.16)^†^**
**Number of new sexual partners (last month)**			0.91 (0.84-0.99)^†^*	0.90 (0.82-0.99)^†^*
**HIV result**				
Negative				1.0
Positive				5.25 (1.95-14.14)**

† = variable use as continuous § = Final model adjusted for education * = p ≤ 0.05 ** = p ≤ 0.01.

## Discussion

In this first epidemiological study of cervical HPV infection in Cambodia, we found that HPV infection was common (41.1%) among women engaged in sex work in Phnom Penh. The prevalence of cervical HPV is comparable to that observed in FSW in Japan (48%)[31], Korea (47%)[23], Tunisia 43.8%[32], Senegal (43%)[33], and Bulgaria (43.4%)[34], but somewhat lower than other locales including Vietnam (85%)[6], Kenya (55.6%)[35], Peru 65.8%[36] and the Philippines (57.2%)[9]. The mean age of FSW in these studies ranged from 24–31 years old, similar to our study (mean 26 years old). Variations in cervical HPV prevalence reported by these studies may be attributable to the use of different HPV assays, differences in FSW populations, or sampling. The high cervical HPV prevalence found among women engaged in sex working Phnom Penh likely reflects increased sexual exposure, that could lead to higher risk of cervical cancer in this vulnerable population.

In this population of Cambodian FSW, HPV-51 was the most prevalent oncogenic genotype, followed by HPV-16. Other dominant HR-HPV genotypes included HPV-52 and HPV-53, while prevalence of HPV-18 was lower. HPV-70, HPV-71 and HPV-81 were the most frequent low-risk genotypes observed. This is consistent with other epidemiological studies conducted in Southeast Asia where infection with oncogenic genotypes such as HPV-51 and HPV-52 was common [6-10]. However, a wide variation in genotype distribution has been observed in different Southeast Asian countries; HPV-16 was most commonly detected in Thailand and Vietnam, but the second most frequent in Indonesia and Philippines [7]. Similar to Cambodia, HPV-18 was the fourth most common oncogenic HPV genotype in normal cytology in Vietnam, and HPV-51 was the most frequently observed genotype in Indonesia [7,37]. This indicates that many HR-HPV genotypes other than HPV-16 and HPV-18 may play important roles in cervical carcinogenesis in Cambodia, and elsewhere in Southeast Asia. Two commercial HPV vaccines targeting only oncogenic HPV-16 and HPV-18 (Gardasil^™^ and Cervarix^™^) have been approved by national regulatory agencies and are currently used in several countries. To date, Cervarix^™^ is the only vaccine that has been approved in Cambodia but access is limited. Our results and those from other epidemiological studies clearly show that currently available vaccines do not cover the most prevalent HR-HPV genotypes found in Cambodia and other Southeast Asian countries. However, some cross-protection with other HPV subtypes has been observed for Cervarix^™^[38,39]. These findings underscore the potential limited impact of existing HPV vaccines on cervical cancer prevention in Southeast Asia, despite the cross protection that has been observed [11].

Consistent with previous studies, women infected with HIV were more likely to be infected with any cervical HPV genotype compared with HIV-negative women. [21,22,40]. Simultaneous infection with multiple cervical HPV genotypes was also higher among women infected with HIV, which is consistent with other studies [35,40]. A possible explanation is that HIV and HPV are both sexually transmitted infections, sharing common modes of transmission. Biological mechanisms involving the immune system might also be implicated; the immune response elicited by HPV infection and associated clearance of HPV infection, could predispose women to HIV acquisition [41-43]. Thus, women in the process of clearing an HPV infection might be at higher risk of HIV infection [44]. Immunosuppression caused by HIV infection can also increase susceptibility to viral acquisition, lead to inability to clear HPV infection or potentiate reactivation of latent HPV infections [18,22]. Persistent cervical HPV infection is a major cause of cervical cancer and the fact that it is more common among HIV-infected women makes primary prevention by immunization prior to acquisition of HPV more crucial. Two recent studies have shown that the quadrivalent vaccine Gardasil^™^ was safe and immunogenic in cohorts of HIV-infected children and men infected with HIV [45,46].

This is the first study to show that recent binge use of ATS is associated with multiple HPV genotypes, even after controlling for sexual behaviors, HIV and STI infection. ATS use has emerged as a potential significant problem among FSW in Cambodia, and Southeast Asia more generally [29,47]. ATS has been associated with incident STI, including HIV among many groups [48,49] including FSW [29,50,51]. ATS users are at higher risk of STIs due to their risky sexual behaviors, but also because of their exposure to social networks with high HIV/STI prevalence. Drug use, especially stimulant use, has also been linked to perturbations of the immune system in both animal models and humans, possibly leading to increased risk of HIV and STI infection [52-56]. A recent study has shown that cocaine use was associated with increased risk of oncogenic and nononcogenic HPV infection among women [57]. The mechanisms by which ATS is associated with increased risk of HPV infection is unknown but the immune response to HPV infection might be altered by the use of stimulants such as cocaine and ATS. HIV-infected women are already immunosuppressed and prone to numerous co infections. ATS use combined with HIV seropositivity could synergistically act on suppression of the immune system and increase susceptibility to HPV infection. Further clinical and laboratory research is needed to better understand the immunomodulatory effects of stimulants on HPV infection and the underlying mechanisms in a context of high HIV prevalence.

Women working as freelance FSW or in brothels were at higher risk of being infected with multiple cervical HPV genotypes. Our group and others have shown that these women are more susceptible to HIV and STI infection compared with their counterparts working in entertainment and service venues [25,58,59]. Sex work environments influence behaviors, and FSW working as freelancers or in brothels may engage in riskier sexual practices, be more likely to use drugs, and have a greater likelihood of having HIV or STI infected partners. In our study, prevalence of any or multiple cervical HPV infection increased with the number of sexual partners reported by FSW, a finding that has been well documented by others [15,16].

Our study has several limitations. The cross-sectional design of this study does not permit us to conclude causality between HPV infection and risk factors studied here with certainty, as experimental design would. Cervical HPV may not necessarily represent total HPV infection, which can also include oral, vaginal/vulvar, and anal HPV. Self-reported data on sexual behaviors and drug use may introduce response bias associated with socially acceptable or desired answers. Measuring condom use with the last partner might not be representative of general condom use behaviors. However, this would result in conservative estimates of risk, biasing our results toward the null. Finally, participants in this study were not sampled probabilistically; as a consequence our rresults may not be generalizable to all young women in sex work in Phnom Penh or Cambodia. Nevertheless, our sample included women from a wide range of sex work venues and captured a wide breadth of this high-risk occupational group in Phnom Penh.

## Conclusions

Our findings highlight the burden of cervical HPV infection among FSW in Phnom Penh and, in particular the vulnerability of women infected with HIV and those using ATS. Routine screening for cervical cancer is not available for most Cambodian women and few if any treatment options exist upon diagnosis with pre-malignant lesions or cervical cancer. These first results on prevalence of cervical HPV infection and the genotypes present among Cambodian women offer important insights into the need for cervical cancer screening strategies and immunization programs in this country. However, more epidemiological research is needed to examine HPV infection at a population level in Cambodia and, in particular to inform the development of effective prevention interventions for young and high-risk women. Paying for sex is widely accepted among men in Cambodia and the number of women engaged in sex work has grown substantially in the past few years; estimated at 34,193 [60]. Finally, our findings underscore the urgent need of for accessible HPV and cervical cancer prevention and treatment, as well as the importance of combining prevention and treatment of drug use with HIV prevention and reproductive health intervention programs targeting FSW in Cambodia.

## Competing interest

The authors declare that they have no competing interests.

## Authors’ contribution

MCC, KP, ESS, NS, KS, JLE, JK, LM and JP designed the YWHS prospective study and contributed to data acquisition. MCC managed the literature search and summaries of previous related studies, conceived the design of the analysis and undertook the statistical analysis. KP, JK, JP and MCC contributed to data interpretation. MCC wrote the first draft of the manuscript and made modifications after comments from the co-authors. All authors contributed with comments and suggestions and have approved the final manuscript.

## Authors’ information

‘On behalf of the Young Women’s Health Study Collaborative (John Kaldor, Serey Phal Kien, Lisa Maher, Tooru Nemoto, Kimberly Page, Joel Palefsky, Vonthanak Sapphon, Mean Chhi Vun).

## Acknowledgments

The authors would like to acknowledge the coordinated efforts and dedication of the research teams at the National Center for HIV/AIDS, Dermatology, and STDs and the Cambodian Women’s Development Agency. We also want to thanks Maria Da Costa from University of California San Francisco and Dr. Soriyann from the National Maternal and Child Health Center (NMCHC) in Phnom Penh for their help with the laboratory analyses. Finally, we are indebted to all the Cambodian women who agreed to participate in this study and grateful for the privilege to work with them. This work was supported by the National Institute of Health “U01AI0154241”, “1R21 DA025441”, and “1R01NR010995”. M-C Couture received financial support from the Canadian Institutes of Health Research (postdoctoral fellowship award). HPV quadrivalent vaccine (Gardasil^T.M^) was generously donated by Merck. The NIH and Merck had no further role in study design; in the collection, analysis and interpretation of data; in the writing and the submission of the paper for publication.

## Pre-publication history

The pre-publication history for this paper can be accessed here:

http://www.biomedcentral.com/1471-2334/12/166/prepub
